# Assessing construct reliability through open-ended survey response analysis

**DOI:** 10.1371/journal.pone.0320570

**Published:** 2025-04-01

**Authors:** Katherine E. Koralesky, Marina A.G. von Keyserlingk, Daniel M. Weary

**Affiliations:** Animal Welfare Program, Faculty of Land and Food Systems, The University of British Columbia, Vancouver, British Columbia, Canada; Paris School of Economics, FRANCE

## Abstract

Online surveys often include quantitative attention checks, but inattentive participants might also be identified using their qualitative responses. We used the software Turnitin™ to assess the originality of open-ended responses in four mixed-method surveys that included validated multi-item rating scales (i.e., constructs). Across surveys, 18-35% of participants (n =  3,771) were identified as having copied responses from online sources. We assessed indicator reliability and internal consistency reliability and found that both were lower for participants identified as using copied text versus those who wrote more original responses. Those who provided more original responses also provided more consistent responses to the validated scales, suggesting that these participants were more attentive. We conclude that this process can be used to screen open-ended responses from online surveys. We encourage future research to replicate this screening process using similar tools, investigate strategies to reduce copying behaviour, and explore the motivation of participants to search for information online, including what sources they find compelling.

## Introduction

Online surveys are commonly used by researchers to examine public views on a variety of topics. As the use of online surveys has grown, so has the research on survey data quality [1,2–4]. For example, research on the advantages and drawbacks of using online platforms has led to the development of recommendations [5,6] and a call for the use of reporting standards [7]. Assessing participant “attention” (or “inattention”) is a common recommendation, and disciplines use terms such as “careless responding”, “inefficient effort responding” or “random responding” to characterize this behaviour [4]; we use the term “attentiveness” in this paper. Much of this research focuses on Amazon Mechanical Turk (MTurk), likely because it is one of the most frequently used platforms [5,8]. MTurk provides a large participant pool, reasonable cost, flexibility regarding research design, accessibility and efficient data collection [5], and can provide quality data [9,10]. However, MTurk is also susceptible to inattentive workers, misrepresentation by workers, workers with inconsistent English language fluency, and vulnerability to code used to automatically complete surveys (i.e., bots) [2,5,11]. Researchers have investigated these concerns and confirmed that the MTurk Approval Rating system is not an effective measure for improving data quality [12] and that MTurk data quality has decreased over time [13]. Platforms like CloudResearch and Prolific can generate higher quality data [14], but are subject to the same challenges.

Researchers have developed specific strategies to improve data quality [2,5,15], including measures of survey completion time as an indirect way to identify inattentive participants [16,17] and bot detection [18]. Attention and manipulation checks are commonly used to identify whether participants have read and understood the questions; these can include asking participants to respond to logical statements and directed queries [19], instructed manipulation checks (i.e., where participants are asked to select a specific response on a scale [20]; and others [21].

Attention checks can improve data quality but also have drawbacks [2,21,22]; for example, checks are sensitive to participant learning, vary in terms of difficulty [2] and can frustrate [19], as well as distract participants and interrupt survey flow [23]. Also, participant attentiveness can vary across survey topics, attention check questions, and within participants at different points in time [21]. Some research has found that attention checks can bias samples based on participant education, age and gender [4]. Attentiveness is likely not a stable participant characteristic, but rather depends on survey context, and some have suggested including multiple attention check questions and stratifying results by attention check response to avoid sampling biases [4].

Analysis of open-ended qualitative responses provides another opportunity for improving data quality. Open-ended responses are common in mixed-method surveys, where researchers typically collect and analyze quantitative and qualitative data [24]. Screening of qualitative responses has been suggested as an additional attention check strategy [5]. Common screening strategies include removing participants who provide responses that cannot be meaningfully interpreted or are nonsensical [13,25]. Additionally, participants who fail to meet criteria for response length or discuss a small number of themes in a response, with fewer themes mentioned indicating a lack of familiarity with the topic, can be removed [26]. Such screening is usually done manually and thus requires additional time to review individual survey responses, as well as researcher assessment which involves subjectivity [19].

We screened qualitative data from two of our studies using the strategies above [27,28], but noticed some similarities across responses. We pasted several responses into the search engine Google and found they were copied word-for-word from online sources. How often participants use online sources to answer MTurk survey questions is unknown [11], and few studies to date have investigated this behaviour. For example [29], analyzed the frequency of participants who self-reported looking for answers to factual political knowledge questions (e.g., current USA unemployment rate) and tested interventions to decrease this behaviour. In another instance [30], detected “search behaviour” by asking participants a very difficult political knowledge question and considered correct answers indicative of search behaviour. Next [31], confirmed that participants commonly looked online for answers to political knowledge questions, and discussed how this behaviour harms survey validity and can lead to misrepresentations of public knowledge. Using a different approach [32], developed TaskMaster, a Javascript tool that can be integrated into Qualtrics surveys and monitors when participants leave the survey window; this tool has been used to confirm self-reported search behaviour on survey questions [12].

The aim of this paper is to describe a novel screening process for open-ended responses in online surveys. We used the online writing tool Turnitin™ [33] to assess the originality of open-ended responses collected in four different public surveys about genetic engineering in farm animals. Turnitin™ is available in many universities, easy to use and detects text that has been copied from online sources. We first describe the frequency and percentage of participants who were identified by Turnitin™ as providing responses copied from online sources, and use a two-sample t-test to compare survey completion time for participants who copied versus those who wrote more original responses. We then describe indicator reliability and internal consistency reliability (using Cronbach’s alpha) of multi-item rating scales for participants who provided copied versus more original responses, and statistically compare Cronbach’s alpha values. We report indicator reliability and internal consistency reliability as our main outcomes to demonstrate that participants who provided copied responses responded less consistently to multi-item rating scales than those who provided more original responses. We conclude with a discussion about the motivation of participants to search for information online when responding to surveys and limitations of the study.

## Method

### Ethics

We assessed our screening process using responses to four different surveys collected in two studies, both approved by the University of British Columbia (UBC) Behavioural Research Ethics Board (Study 1 # H21-00047 and Study 2 # H22-00873).

### Participant recruitment

We created surveys in Qualtrics (Provo, UT, USA) and used MTurk to recruit participants from the USA who were 18 years of age or older (see Supplemental Materials for a copy of each survey; https://doi.org/10.5683/SP3/PEPATK). Participant quotas were set based on representative data from the United States Census Bureau [34]. We ran each survey at different times during 2021 and 2022, beginning on June 10, 2021 and ending on May 24, 2022. This recruitment resulted in a convenience sample of MTurk participants who were willing to take the survey when it was available. Participation was anonymous and confidential and each participant provided written consent to take the survey. We compensated participants $1 USD.

### Survey design and measures

This study uses open-ended qualitative data from four different surveys. In Study 1, we conducted three separate surveys (henceforth referred to as Study 1, Surveys 1, 2 and 3) and asked participants to describe their understanding of genetic engineering technology terms (e.g., gene editing, genetic modification; Surveys 1, 2) and the perceived acceptability of genetic engineering in farm animals (Survey 3; [27]; see Supplemental Materials; https://doi.org/10.5683/SP3/PEPATK). In Study 2, we conducted one survey (henceforth referred to as Study 2, Survey 4) soliciting top-of-mind public questions about gene editing in farm animals ([28]; see Supplemental Materials; https://doi.org/10.5683/SP3/PEPATK).

Each survey also included validated multi-item rating scales that typically achieve high levels of internal consistency and are used to create a construct (e.g., trust). In Study 1 [27] we used a causal trust-acceptability model involving four constructs [35,36] previously used to assess social acceptance of novel technologies [e.g., 36,37]. Each construct includes individual indicator variables, including the construct of acceptance (4 variables), benefit (3 variables), risk (3 variables), and social trust (5 variables). In Study 2 [28], we used several scales: the Aversion to Tampering with Nature (ATN, which includes five variables; [38]), benefit, risk and attitude scales (each including 3 variables; [39] and a social trust in institutions scale (4 variables; [35].

### Data preparation and analysis

Each survey used the same three data quality screening procedures (full details are provided in each study), removing: 1) incomplete responses, 2) responses from participants who failed the instructed manipulation check (participants were asked to select a specific response on a scale) [20], and 3) responses from participants who did not respond to the qualitative question or who provided non-sensical text (e.g., “nice animals”, “engineered animals are numerals”; [26]).

To systematically determine if responses were copied, we submitted all remaining open-ended responses to Turnitin™ using the Quick Submit function. We selected search repositories available through UBC’s Turnitin™ license, including the internet, student papers and periodicals, journals and publications. We found that most responses were copied nearly word-for-word from online sources, indicated by Turnitin™ highlighting the entire response; categorizing these responses as copied was straightforward. However, some responses had only a portion of the text highlighted, for example when phrases like “the genome of an organism” or “DNA being modified or edited” were used. For responses with a portion of text highlighted, we categorized those with more than 50% of the content identified as similar to online sources as “mostly copied” (for ease, henceforth referred to as “copied”), and responses with less than 50% of the content identified as similar to online sources as “not mostly copied” (for ease, henceforth referred to as “not copied”).

We could not conduct a-priori power analyses because we were unaware participants would perform search behaviour and look for information online. In this study, we analyzed data descriptively and used different procedures to assess statistical differences, and report effect size where possible. First, in each group (copied and not copied) for each of the four surveys, we categorized participant demographic information and performed two-sample t-tests to compare mean survey completion times for copied and not copied groups and calculated effect size using Cohen’s d. We then assessed the reliability of constructs by determining indicator reliability and internal consistency reliability [40]. Indicator reliability measures the correlation between a construct and the individual indicator variables (i.e., questions). To assess indicator reliability, we used PROC CALIS in SAS ® OnDemand for Academics (version 9.4, SAS Institute Inc.) to assess model fit for each construct and the corresponding indicator variables using a measurement model. We measured indicator reliability for each construct in each group (copied and not copied), for each of the four surveys. This procedure generates a t-value for each indicator variable to validate the relationship between the indicator variable and construct; significant t-values confirm the validity of the measurement model. Indicator reliability estimates above 0.7 are recommended, loadings between 0.4 and 0.7 should be considered for removal, and loadings below 0.4 should be removed [40].

To assess internal consistency reliability, we used Cronbach’s alpha. Internal consistency reliability measures the degree of reliability (i.e., interrelatedness of indicator variables) for each construct [40]. We measured internal consistency reliability for each group (copied and not copied), for each of the four surveys. Cronbach’s alpha can range from 0 to 1, with values from 0.7 to 0.95 generally considered acceptable although the value is also a function of the number of items in a scale [41]. We statistically compared Cronbach’s alpha values using the cocron web interface [42], a platform-independent R package that estimates a χ2 distribution based on the hypothesis that values are statistically different.

## Results

Our first aim was to describe how often participants provided copied responses. The four surveys considered a total of 3,771 participants, 837 of whom were identified as providing responses copied from online sources. Across surveys, 18–35% of participants provided content identified as at least 50% copied: Study 1, Survey 1: 18%; Study 1, Survey 2: 18%; Study 1, Survey 3: 24%; Study 2, Survey 4: 35%. Table 1 provides the exact open-ended question asked and demographic information about the participants in each survey, separately for those who provided content identified as mostly copied versus those who did not. Generation was categorized following [43] and ages were adjusted to reflect the data collection date: Generation Z includes participants ≤  24 years of age, Millennial includes participants aged 25–40 and Pre-millennial includes participants aged ≥  41 years of age.

**Table 1:** Descriptive statistics about survey participants in each survey who provided responses identified as copied, or not identified as copied and the total number of responses and frequencies for each demographic, including Generation, Gender and Education.

Demographic	How do you understand the term gene editing?^a^			How do you understand the term [technology term]?^b^			In a few sentences, please describe how acceptable you think this technology is for the [animals or plants depending on treatment] involved.^c^			What questions come to mind about gene editing animals?^d^		
	Copied	Not Copied	Total	Copied	Not Copied	Total	Copied	Not Copied	Total	Copied	Not Copied	Total
Generation												
Pre-millennial ≥ 41 years	79 (14%)	488 (86%)	567	78 (19%)	342 (81%)	420	87 (15%)	494 (85%)	581	100 (39%)	156 (61%)	256
Millennial = 25-40	115 (24%)	372 (76%)	487	80 (22%)	292 (78%)	372	107 (29%)	268 (71%)	375	48 (24%)	150 (76%)	198
Generation Z ≤ 24	20 (14%)	121 (86%)	141	11 (8%)	132 (92%)	143	78 (46%)	93 (54%)	171	31 (54%)	26 (46%)	57
Total	214	981	1195	169^f^	766	935^f^	272^g^	855	1127^g^	179	332	511
Gender^h^												
Woman	76 (14%)	484 (86%)	560	95 (17%)	453 (83%)	548	143 (25%)	435 (75%)	578	72 (35%)	135 (65%)	207
Other	135 (21%)	493 (79%)	628	75 (19%)	312 (81%)	387	129 (24%)	418 (76%)	547	107 (35%)	197 (65%)	304
Total	211^e^	977^e^	1188 ^e^	170^f^	765^f^	935^f^	272^g^	853^g^	1125^g^	179	332	511
Education												
Did not finish high school	0	9 (100%)	9	1 (25%)	3 (75%)	4	1 (50%)	1 (50%)	2	n/a^i^		
High school graduate	2 (2%)	81 (98%)	83	6 (8%)	68 (92%)	74	10 (14%)	64 (86%)	74			
Some college, no degree	10 (4%)	214 (96%)	224	5 (3%)	149 (97%)	154	6 (4%)	136 (96%)	142			
Trade degree	7 (11%)	55 (89%)	62	4 (9%)	42 (91%)	46	1 (2%)	57 (98%)	58			
Bachelor degree	152 (24%)	475 (76%)	627	114 (23%)	372 (77%)	486	187 (30%)	440 (70%)	627			
Graduate degree	43 (23%)	147 (77%)	190	41 (24%)	132 (76%)	173	68 (30%)	157 (70%)	225			
Total	214	981	1195	171	766	937	273	855	1128			

^a^Study 1, Survey 1; no length requirement for responses.

^b^Study 1, Survey 2; no length requirement for responses.

^c^Study 1, Survey 3; length requirement of 60 characters (not including spaces).

^d^Study 2, Survey 4; one question required. We used two additional data screening strategies: 1) responses (n = 10 and n = 2) flagged by Qualtrics as duplicates were removed from the copied and not identified as copied groups, respectively; and 2) responses (n = 11 and n = 6) flagged as invariant were removed from the copied and not identified as copied groups, respectively.

^e^For Gender, 7 missing values (3 copied, 4 not copied).

^f^For Gender, 2 missing values (1 copied, 1 not copied) and for Generation, 2 missing values (2 copied).

^g^For Gender, 3 missing values (1 copied, 2 not copied) and for Generation, 1 missing value (1 copied).

^h^For Gender, participants could select from the following options: Woman, Man, Transgender woman, Transgender man, Non-binary, Two-spirit, Not-listed, Prefer not to answer. We categorized Gender in two categories for descriptive statistical analysis: 1) woman and 2) all other selections. We included the frequency of all participants in Study 2, Survey 4, which included 1 Transgender woman, 8 Transgender men, 2 Non-binary and 1 Prefer not to answer.

^i^We did not collect demographic information about Education in Study 2, Survey 4.

Using two-sample t-tests, we found no difference in completion times between participants identified as having copied responses versus those not identified as having copied responses for Surveys 1 and 3 in Study 1: Mean ±  SE completion times were 5 min 27 s ±  22 s vs. 5 min 18 s ±  12 s (t_1193_ =  −0.31, p =  0.75; d =  0.02), and 5 min 8 s ±  17 s vs. 5 min 37 s ±  10 s (t_1126_ =  1.42, p =  0.16; d =  0.10) for copied and not copied responses in these two surveys, respectively. In the other two surveys we found differences but these were inconsistent; participants who copied took longer to complete Survey 2 in Study 1: 5 min 19 s ±  23 s vs. 4 min 26 s ±  9 s (t_935_ =  −2.52, p =  0.01; d =  0.21). In Study 2, those who copied completed the survey more quickly than those who did not: 6 min 24 s ±  19 s vs. 8 min 5 s ±  22 s (t_526_ =  2.96, p =  0.003; d =  0.27).

Our second aim was to describe indicator reliability and internal consistency reliability of each construct in each survey for participants who provided copied versus more original responses. In Study 1, Surveys 1, 2, and 3, for the constructs of Acceptance, Benefit and Risk, each indicator reliability estimate for the participants who copied had weaker associations compared to the participants who provided more original responses (Table 2). For Social Trust, in Study 1, Survey 1 and 2, estimates were stronger for each indicator in the not copied group with the exception of T5 in Surveys 1 and 2, and T3, T4 and T5 in Survey 3. In those cases, indicator estimates were stronger for the participants who copied versus those who did not copy. In Study 2, Survey 4, all indicators for participants who copied fell within the threshold to be considered for removal (i.e., 0.4–0.7); indicators for those who provided more original responses were above the threshold for acceptance ( > 0.7; Table 3).

**Table 2:** Standardized indicator reliability estimates for Study 1, Survey 1, 2 and 3 respectively (S1–S3). Estimates are shown for each indicator for each of four constructs examined (Acceptance, Benefit, Risk, Social Trust), and for participants who provided responses identified as copied and those whose responses were not identified as copied. All indicator reliability estimates are significant (p <  0.001).

		S1_S1 Copied n = 214	S1_S1 Not Copied n = 981	S1_S2 Copied n = 171	S1_S2 Not Copied n = 766	S1_S3 Copied n = 273	S1_S3 Not Copied n = 855
Construct	Indicators	Factor loadings					
Acceptance	A1	0.50	0.86	0.55	0.87	0.72	0.88
	A2	0.64	0.87	0.58	0.85	0.59	0.84
	A3	0.51	0.89	0.67	0.88	0.79	0.89
	A4	0.48	0.89	0.54	0.86	0.68	0.85
Benefit	B1	0.59	0.82	0.61	0.81	0.75	0.80
	B2	0.63	0.81	0.63	0.82	0.62	0.78
	B3	0.48	0.84	0.70	0.79	0.64	0.81
Risk	R1	0.71	0.92	0.76	0.92	0.80	0.92
	R2	0.51	0.82	0.51	0.79	0.57	0.78
	R3	0.68	0.77	0.76	0.81	0.75	0.77
Social Trust	T1	0.73	0.90	0.72	0.85	0.83	0.89
	T2	0.68	0.91	0.76	0.90	0.81	0.88
	T3	0.79	0.88	0.82	0.85	0.86^a^	0.86
	T4	0.75	0.83	0.74	0.75	0.84^a^	0.82
	T5	0.79^a^	0.68	0.85^a^	0.63	0.87^a^	0.70

^a^In these cases, indicator estimates were stronger for the participants who copied versus those who did not copy. All other indicator estimates were stronger for the participants who were in the not copied group.

**Table 3:** Standardized indicator reliability estimates for Study 2, Survey 4. Estimates are shown for each indicator for each of five constructs examined (Attitude, Benefit, Risk, Social Trust, ATN), and for participants who provided responses identified as copied and those whose responses were not identified as copied. All indicator reliability estimates are significant (p <  0.001).

		Copied n = 179	Not Copied n = 332
Construct	Indicators	Factor loadings	
Attitude	A1	0.63	0.87
	A2	0.57	0.88
	A3	0.45	0.81
Benefit	B1	0.65	0.86
	B2	0.60	0.89
	B3	0.69	0.89
Risk	R1	0.54	0.83
	R2	0.58	0.88
	R3	0.63	0.77
Trust	T1	0.43	0.74
	T2	0.56	0.85
	T3	0.51	0.90
	T4	0.44	0.90
ATN	AT1	0.56	0.72
	AT2	−0.51	−0.10
	AT3	0.63	0.77
	AT4	0.57	0.74
	AT5	0.57	0.79

The internal consistency reliability of all constructs was lower for participants who copied responses to open-ended questions versus those who provided text that was not copied (Tables 4, 5). This finding held across all four surveys included in the current study. We compared Cronbach’s alpha values and found that all but two were statistically different between the copied and not copied respondents. Consistent with the indicator reliability estimates reported above, there was no statistical difference for the Social Trust construct in Study 1, Survey 2 and Survey 3 between the copied and not copied groups.

**Table 4:** Cronbach’s alpha and 99% confidence intervals for Study 1, Surveys 1, 2 and 3. Values and confidence intervals are shown separately for each of four constructs examined (Acceptance, Benefit, Risk and Social Trust), and for participants who provided responses identified as copied and those whose responses were not identified as copied. For each of the constructs Cronbach’s alpha was significantly higher (p <  0.01) for participants who provided the more original responses, with the exception of the Social Trust construct for Surveys 2 and 3.

Construct	Survey 1		Survey 2		Survey 3	
	Copied n = 214	Not Copied n = 981	Copied n = 171	Not Copied n = 766	Copied n = 273	Not Copied n = 855
Acceptance	0.61 [0.48, 0.71]	0.93 [0.92, 0.94]	0.68 [0.56, 0.77]	0.92 [0.91, 0.93]	0.79 [0.73, 0.84]	0.92 [0.91, 0.93]
Benefit	0.58 [0.43, 0.69]	0.86 [0.84, 0.88]	0.68 [0.55, 0.77]	0.84 [0.81, 0.86]	0.70 [0.61, 0.77]	0.84 [0.81, 0.86]
Risk	0.66 [0.54, 0.75]	0.87 [0.85, 0.89]	0.69 [0.57, 0.78]	0.87 [0.85, 0.89]	0.74 [0.66, 0.80]	0.86 [0.84, 0.88]
Social Trust	0.86 [0.82, 0.90]	0.92 [0.91, 0.93]	0.88 [0.84, 0.91]	0.90 [0.88, 0.91]	0.92 [0.90, 0.94]	0.92 [0.91, 0.93]

**Table 5:** Cronbach’s alpha and 99% confidence intervals for Study 2, Survey 4. Values and confidence intervals are shown separately for each of five constructs examined (Attitude, Benefit, Risk, Social Trust, and ATN), and for participants who provided responses identified as copied and those whose responses were not identified as copied. The indicator AT2 was removed due to poor correlation with the ATN construct. For each of the constructs Cronbach’s alpha was significantly higher (p <  0.01) for participants who provided the more original responses.

	Study 2	
Construct	Copied n = 179	Not Copied n = 332
Attitude	0.58 [0.42, 0.70]	0.89 [0.86, 0.91]
Benefit	0.69 [0.57, 0.78]	0.91 [0.89, 0.93]
Risk	0.62 [0.47, 0.73]	0.87 [0.83, 0.90]
Social Trust	0.55 [0.39, 0.68]	0.91 [0.89, 0.93]
ATN	0.69 [0.58, 0.78]	0.84 [0.80, 0.87]

## Discussion

We assessed responses of 3,771 participants, recruited to participate in four different surveys and responding to quantitative scales used to develop nine different constructs, and found that most indicators and all constructs were less reliable for participants who provided text identified as copied than participants who provided more original responses. In all but two comparisons, Cronbach’s alpha values were significantly higher when considering only participants who provided more original responses. It is important to note that our analysis considered data from participants who had passed the instructed manipulation check [20] in the surveys and who wrote coherent responses. We found no consistent evidence that survey completion time varied in relation to the provision of copied text; this result agrees with previous work suggesting that survey completion time is not sensitive or specific for identifying participants who may be less engaged with the survey [4]. Research has found that most online survey participants tend to pass attention check questions, and thus there may be a need for more nuanced data quality control strategies [1,44]. Our screening process can provide nuanced data quality control and responds to calls to incorporate open-ended response analysis to screen data [5].

We found that Cronbach’s alpha values were higher for participants who provided more original responses. Inattentive participants often do not answer validated scales consistently [15]. We considered that the responses of participants who copied were of lower quality than those who wrote more original responses because our aim was to analyze public viewpoints. However, it is possible that for some participants copying was a thoughtful attempt to provide a well-researched response that in some cases may be more valuable than original but top-of-mind responses. Authors [29] found some evidence that participants displayed a form of socially desirable responding called self-deceptive enhancement, where, in the case of taking online surveys, people believe they know something because they know where to find the information online. In our surveys, participants may have misunderstood survey instructions, lacked familiarity with the survey format, or sought information about a topic (genetic engineering) for which they had limited technical knowledge and found it difficult to answer the questions. For some of these individuals, copying may have been their attempt to engage with the survey, rather than disengage. In future surveys, researchers might consider using strategies that have been shown to reduce online searching, including simply asking participants to avoid online searches [29,30].

We found it surprising that participants copied responses even though we specifically asked that they provide their views and top-of-mind responses and questions. It is not clear why participants would choose to copy answers from online sources; time spent looking for answers (and not completing additional surveys) is an opportunity cost for MTurk workers [29]. One hypothesis is that these participants found it easier to copy text than to compose their own response. This aligns with [45], who surveyed UK workers (i.e., people taking surveys) from three large online survey platforms, and reported workers felt ambivalent about completing tasks they described as “neither work nor leisure”. That said, participants who simply wanted to receive payment for the survey could input any response, even one-word or incoherent responses. We think it is unlikely these responses were from bots that automatically completed surveys. Another hypothesis is that these responses were from participants who took the time to search for related answers and copied this material. In this case these responses would reflect what sources people identify when searching for related information, and if there are common sources of information. These responses could potentially identify what arguments and evidence people find compelling. Future work might explicitly direct participants to search for materials that they find compelling or helpful in increasing their understanding of a topic, turning what otherwise might be considered as copying into data on the sources people turn to when considering substantive issues.

In our surveys, 18 to 35% of participants provided responses we flagged as copied. We are unsure as to why this range exists. MTurk data quality has decreased over time [13] and we conducted Study 2, Survey 4 (35% of participants provided copied responses) approximately one year after data collection for Study 1, Surveys 1, 2 and 3. All four surveys included multi-item rating scales and two open-ended questions each, and were approximately the same length. Others have reported similar rates of participants searching for answers to survey questions online [30,31]. Across 30 datasets, [19] reported average participant losses from different forms of attention checks: 14% (directed queries), 18% (logical statements) and 20% (manipulation checks). While removing these participants improved construct and scale fit [19], removing participants to improve data quality must be done with caution. Removal can result in loss of power (due to reduced sample size), or increased power (due to reduced variation within treatment); how these two factors vary will depend upon the specifics of the case. Further, removing participants can disproportionately exclude certain demographic characteristics (e.g., age, gender, education) and further induce bias [4]. We did not note any obvious relationship with participant age or gender, and time to complete the surveys, but there appeared to be some association with education level. Further work may wish to follow up on these results.

While our surveys focused on perspectives about the use of emerging biotechnologies like genetic engineering in animals, screening open-ended responses using Turnitin™ may be generalizable to other topics, for example research investigating public engagement with science and technology. This research has evolved over time [46], and scholars are now rethinking how to design engagement exercises that involve publics more deliberately, shifting away from a one-way flow of information from experts to members of the public [47]. Our screening process identified how some survey participants engaged with the topic on their own by searching for information online. Further research on a range of topics, both technical and familiar, is required to better understand the motivation and methods used for searching for online content.

Categorizing participants as “inattentive” or “careless” is likely oversimplified. Determining participant attentiveness is nuanced, leading to recommendations for transparent reporting of screening measures [4]. In addition to reporting screening measures in this paper, we avoided terms like “inattentive” to describe our sample, and instead described participants as those who copied and those who wrote original responses. Future work might assess construct reliability of participants removed using our screening process versus participants removed using common quantitative attention checks (e.g., logical statements, instructed manipulation checks). As well, our screening process could be used to determine if participants who fail common quantitative attention checks are more likely to provide copied responses.

Adding a survey question to assess participant knowledge may have helped identify participants who copied due to a lack of knowledge versus those who copied due to inattentiveness. Assessing objective or subjective knowledge about genetic engineering technology in public opinion surveys is common, but these assessments are predetermined by researchers and likely miss some types of knowledge participants have [28]. Asking participants what they want to know can provide information about public understanding on a topic, and which topics they want to know more about [28].

Our study has limitations. MTurk has a large participant pool [5], but we acknowledge that our samples were not representative. We found that all but two Cronbach’s alpha values were statistically different between the copied and not copied group, but the procedure we used does not report effect size [42]. We did not include additional qualifications for MTurk workers (e.g., a Human Intelligence Task Approval Rating), although this qualification is not always effective for controlling data quality [12]. While most responses were copied word-for-word, in cases where Turnitin™ highlighted part of the text as copied, we categorized responses as copied or not copied using a 50% threshold. Others may wish to classify responses in a different way or assess different thresholds. Turnitin™ may be unable to detect the use of artificial intelligence like ChatGPT to generate survey responses. We encourage other researchers to replicate our screening process to assess the originality of open-ended survey responses using other tools (e.g., iThenticate) and platforms (e.g., CloudResearch) to further develop this screening method.

## Conclusion

We used Turnitin™ to categorize open-ended qualitative responses from four different surveys about genetic engineering. Most indicators and each construct in each survey were less reliable for participants who provided copied responses versus those who provided more original responses. We encourage researchers who use mixed methods or conduct surveys with open-ended questions to use this or similar tools to identify such participants, and to consider removing these from their dataset, or analyzing these responses separately. Future work should also investigate what sources of information are preferentially used by participants, and the reasons why people choose to copy rather than write original responses.
